# Supplementary material for: Optimizing EEG Source Reconstruction with Concurrent fMRI-Derived Spatial Priors
Source: Brain Topogr. 2022 Feb 10;35(3):282–301. doi: 10.1007/s10548-022-00891-3 (PMC9098592; doi:10.1007/s10548-022-00891-3)
Supplement: Supplementary file 1 — Supplementary file1 (DOCX 52 KB) [file 10548_2022_891_MOESM1_ESM.docx]

**Title**

Optimizing EEG source reconstruction with concurrent fMRI-derived spatial priors

**Authors**

Rodolfo Abreu^1,a,#^, Júlia F. Soares^1,b,#^, Ana Cláudia Lima^2^, Lívia Sousa^2,3^, Sónia Batista^2,3^, Miguel Castelo-Branco^1,3,c^, João Valente Duarte^1,3,d,*^

**Affiliations**

^1^ Coimbra Institute for Biomedical Imaging and Translational Research (CIBIT), Institute for Nuclear Sciences Applied to Health (ICNAS), University of Coimbra, Coimbra, Portugal

^2^ Neurology Department, Centro Hospitalar e Universitário de Coimbra, Coimbra, Portugal

^3^ Faculty of Medicine, University of Coimbra, Coimbra, Portugal

^#^ Authors contributed equally to this work

a ^
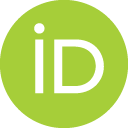
^ <https://orcid.org/0000-0001-9496-3534>

b ^
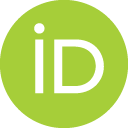
^ <https://orcid.org/0000-0002-8960-7794>

c ^
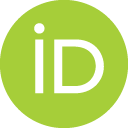
^ <https://orcid.org/0000-0003-4364-6373>

d ^
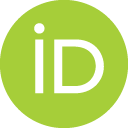
^ <https://orcid.org/0000-0001-8586-9554>

* *Corresponding author*

Email address: joao.v.duarte@fmed.uc.pt

Phone number: +351 239 488510

**Acknowledgments**

We would like to thank the participants for their involvement in this study. We are also very grateful to Sónia Afonso and Tânia Lopes for the help with MRI setup and scanning.

###### Supplementary Material

|  | **Number of lesions with objective clinical evidence** | **Additional data needed for a diagnosis of multiple sclerosis** |
| --- | --- | --- |
| ≥ 2 clinical attacks | ≥ 2 | None* |
| ≥ 2 clinical attacks | 1 (as well as clear-cut historical evidence of a previous attack involving a lesion in a distinct anatomical location†) | None* |
| ≥ 2 clinical attacks | 1 | Dissemination in space demonstrated by an additional clinical attack implicating a different CNS site or by MRI‡ |
| 1 clinical attack | ≥ 2 | Dissemination in time demonstrated by an additional clinical attack or by MRI§ OR demonstration of CSF-specific oligoclonal bands¶ |
| 1 clinical attack | 1 | Dissemination in space demonstrated by an additional clinical attack implicating a different CNS site or by MRI‡ AND  Dissemination in time demonstrated by an additional clinical attack or by MRI§ OR demonstration of CSF-specific oligoclonal bands¶ |
| If the 2017 McDonald Criteria are fulfilled and there is no better explanation for the clinical presentation, the diagnosis is multiple sclerosis. If multiple sclerosis is suspected by virtue of a clinically isolated syndrome but the 2017 McDonald Criteria are not completely met, the diagnosis is possible multiple sclerosis. If another diagnosis arises during the evaluation that better explains the clinical presentation, the diagnosis is not multiple sclerosis. An attack is defined in panel 1. *No additional tests are required to demonstrate dissemination in space and time. However, unless MRI is not possible, brain MRI should be obtained in all patients in whom the diagnosis of multiple sclerosis is being considered. In addition, spinal cord MRI or CSF examination should be considered in patients with insufficient clinical and MRI evidence supporting multiple sclerosis, with a presentation other than a typical clinically isolated syndrome, or with atypical features. If imaging or other tests (eg, CSF) are undertaken and are negative, caution needs to be taken before making a diagnosis of multiple sclerosis, and alternative diagnoses should be considered. †Clinical diagnosis based on objective clinical findings for two attacks is most secure. Reasonable historical evidence for one past attack, in the absence of documented objective neurological findings, can include historical events with symptoms and evolution characteristic for a previous inflammatory demyelinating attack; at least one attack, however, must be supported by objective findings. In the absence of residual objective evidence, caution is needed. ‡The MRI criteria for dissemination in space are described in panel 5. §The MRI criteria for dissemination in time are described in panel 5. ¶The presence of CSF-specific oligoclonal bands does not demonstrate dissemination in time per se but can substitute for the requirement for demonstration of this measure. | | |

**Table S1:** *The 2017 McDonald criteria for diagnosis of multiple sclerosis in patients with an attack at onset*. (Thompson et al. 2018)
